# Supplementary figures and images for: Allosteric mechanism of signal transduction in the two-component system histidine kinase PhoQ
Source: eLife. 2021 Dec 14;10:e73336. doi: 10.7554/eLife.73336 (PMC8719878; doi:10.7554/eLife.73336)

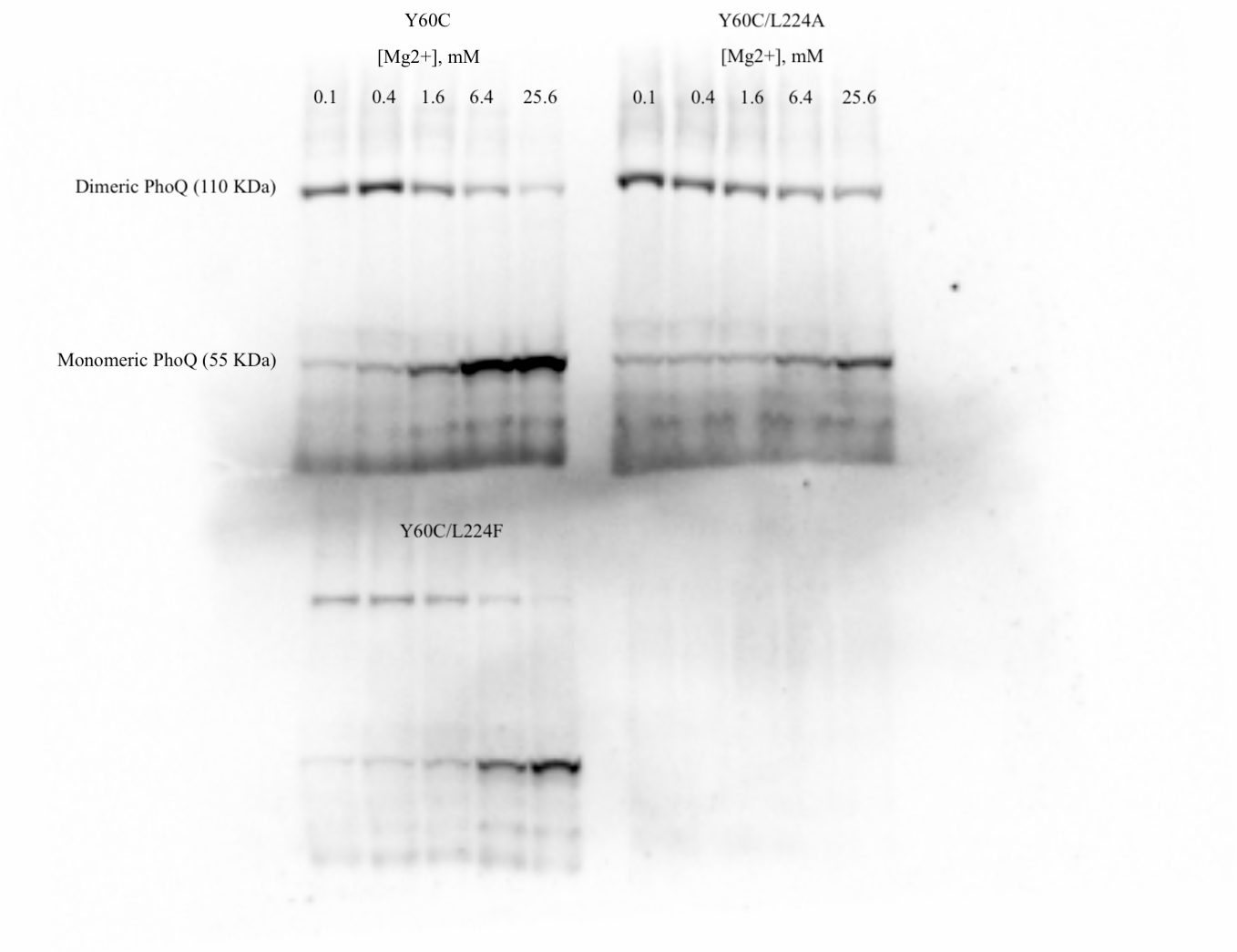

Supplement: Figure 2—figure supplement 1—source data 1. [file elife-73336-fig2-figsupp1-data1.zip › Figure 2-figure supplement 1 Source data/Y60C, L224A crosslinking.tif]
